# Hybrid Cements with ZnO Additions: Hydration, Compressive Strength and Microstructure

**DOI:** 10.3390/molecules27041278

**Published:** 2022-02-14

**Authors:** Magnolia Soto-Felix, Francisco Javier Baldenebro-Lopez, Caleb Carreño-Gallardo, Jose Martin Herrera-Ramirez

**Affiliations:** 1 Departamento de Posgrado de la, Facultad de Ingeniería Culiacán, Universidad Autónoma de Sinaloa, Culiacán 80010, Mexico; msotofelix@uas.edu.mx; 2 Facultad de Ingeniería Mochis, Universidad Autónoma de Sinaloa, Los Mochis 81223, Mexico; francisco.baldenebro@uas.edu.mx; 3 Centro de Investigación en Materiales Avanzados (CIMAV), Laboratorio Nacional de Nanotecnología, Chihuahua 31136, Mexico

**Keywords:** ZnO, sodium silicate, hybrid cements, microstructure, compressive strength

## Abstract

The effect of ZnO has already been studied for Portland cement, but the study of its impact on hybrid pastes is scarce. Thus, in this investigation, the influence of ZnO addition on hydration, compressive strength, microstructure, and structure of hybrid pastes is presented. The analyses were made by setting time tests, compressive strength tests, X-ray diffraction, Fourier-transform infrared spectroscopy, thermogravimetric analysis with differential scanning calorimetry, and scanning electron microscopy coupled with energy-dispersive X-ray spectroscopy. The results indicate that the setting time of the cements was delayed up to 39 min with additions of 3 wt% ZnO. Alternatively, the higher values of compressive strength were observed when 0.5 wt% ZnO was added to the cements for all curing days. In addition, no important differences in the microstructure of samples with different additions of ZnO were observed after 28 days of curing. It is expected that the use of ZnO contributes to the delay of the setting time and the increase of the compressive strength without negatively modifying the microstructure of hybrid pastes.

## 1. Introduction

Portland cement (PC) has been effectively replaced by supplementary cementitious materials (SCM), such as slag, fly ash, silica fume, and natural or artificial pozzolans such as metakaolin, leading to significant improvements of cement properties mainly in mechanical and durability properties [1,2,3,4,5,6,7,8,9]. In addition, this has contributed to sustainability [10,11] due to the replacement of cement, significantly reducing environmental problems related to cement production mainly associated with the calcination of raw materials and the burning of fuels to achieve high temperatures inside a kiln [12,13]. 

However, high replacement ratios in cement are known to be detrimental to mechanical properties development mainly at early ages [14,15]. The early reactivity of SCM can be improved in some ways, such as reducing particle size [16,17], mechanical activation [18,19], thermal activation of natural pozzolans [20,21], and with the addition of chemical activators such as NaOH, KOH, Na_2_CO_3_, or Na_2_SiO_3_, among others [22,23]. Nevertheless, the hydration of PC with highly alkaline solutions has been shown to alter its normal hydration process, with considerable delays in the formation of the main reaction products and the appearance of new products as sodium calcium carbonate and sodium sulfate [24,25]. 

The combination of traditional alkali activation precursors with PC is commonly referred to as hybrid alkali cements [26,27]. This type of cements combines some of the advantages of traditional PC, such as setting at ambient temperature with the development of high early-age mechanical strength and low heat of hydration by the use of alkaline activation of blast furnace slag and fly ash. However, these materials may pose problems of availability and uniformity, which can be resolved with the use of clays in their place [28,29]. In this type of system, the principal hydration products are: C-S-H from the PC and N-A-S-H from the fly ash or metakaolin. In their mutual presence, these gels tend to evolve, respectively, into C-A-S-H and (N,C)-A-S-H [30,31,32].

The study of hybrid cements is not new; many researchers have studied important aspects of hybrid cements. Batuecas et al. [33] examined the compressive strength and porosity of hybrid systems, both from slag and fly ash, in order to determine if the mechanical properties of hybrid and alkali-activated mortars are, indeed, comparable to those from Ordinary Portland Cement (OPC). Results revealed that the assessed alkali-activated material and two hybrid systems case studies obtained mechanical properties comparable to those for OPC.

Qu et al. determined the mineralogical and microstructural changes taking place in a hybrid alkaline cement exposed to high temperatures and the mechanical strength was determined after cooling. The results showed that the hybrid cement pastes had higher residual strength than the reference PC and better post-thermal performance after reaching temperatures of over 800 °C. The above was attributed to the recrystallization of scantly hydraulic phases, such as gehlenite and rankinite [34].

Fernandez et al. focused on sodium sulfate as a possible chemical activator in hybrid cements containing 50% fly ash. The primary aim was to determine whether the manner in which sodium sulfate was added (pre-grinding the solid with the fly ash or dissolving the compound in the mixing water), had a significant effect on mortar mechanical strength development, setting times, heat of hydration, or the nature of the reaction products [35].

Barboza et al. synthesized hybrid cements, reducing the clinker content of PC and used metakaolin and fly ash as SCM in different proportions, which were alkaline-activated with a mixture of sodium silicate and sodium hydroxide. The results indicated that the hybrid cements have similar mechanical properties than OPC, resulting in a dense matrix of hydration products similar to those generated by cements and geopolymers [36]. 

Xue et al. provides an overview on the hydration mechanisms of hybrid alkali cements and durability, including resistance to aggressive solutions and high temperatures, carbonation and efflorescence, chloride ion penetration, and alkali-aggregate reaction. It is evident that this type of cement is more durable than Portland cement in a number of environments; however, the lack of long-term track records in the field is the barrier for application. This work also discusses the knowledge gap to facilitate the future research and development of HAC materials [37].

Moreno et al. explored the application of a new gamma spectrometric method for measuring radionuclide activity in hybrid alkali-activated cements from solid 5 cm cubic specimens rather than powder samples. The research involved assessing the effect of significant variables, such as the nature of the alkaline activator, reaction time and curing conditions, to relate the microstructures identified to the radiological behavior observed. The findings showed that varying the inputs generated pastes with similar reaction products (C-S-H, C-A-S-H, and (N,C)-A-S-H) but different microstructures. The variables involved in hybrid cement activation were shown to have no impact on specimen radioactive content [38]. 

Recently, a new study of hybrid cement with photocatalytic and bactericidal properties based on ceramic tile waste added with ZnO showed the possibility of using constructions and demolition tile waste in high proportions for the elaboration of new rendering mortar added with alkaline solutions and ZnO with innovative properties, such as photocatalytic and bactericidal properties [39]. As can be seen, hybrid cements have been subject to numerous investigations for different applications, evaluating features such as mechanical and thermal properties, and even more complex approaches such as reaction mechanisms. However, the effect of the addition of ZnO on cement has been extensively studied mainly on the mechanisms governing the hydration in PC [40,41,42,43,44]. A few studies on alkali-activated cements have been done [45], but none of these works has focused on the effect of hydration retarders for these types of cements, which could be important for structural and many other applications. 

In this research, the effect of ZnO in hybrid cements was studied using setting time and compressive strength tests, X-Ray Diffraction (XRD), Fourier Transform Infrared Spectroscopy (FTIR), Thermogravimetric Analysis with Differential Scanning Calorimetry (TGA-DSC), Scanning Electron Microscopy (SEM), and Energy-Dispersive X-Ray Spectroscopy (EDX). The results are explained in terms of changes in important variables, such as setting time, compressive strength, and microstructural and structural evolution.

## 2. Results and Discussion

### 2.1. Setting Time 

Table 1 shows the initial and final setting times of samples with additions of 0, 0.5, 1 and 3 wt% ZnO. Setting time of pastes increased with the increase of the ZnO content. The maximum delay of the initial and final setting time with respect to the paste with no ZnO (S0Z) was 22 and 39 min, respectively. These results suggest that the use of SCM in alkali medium significantly reduced the setting times of pastes, due to the accelerating effect of sodium silicate [46].

The accelerating effect of sodium silicate to the mixture is attributed to the loss of consistency of the pastes and the rapid growth of the hydration products, mainly those associated with MK, whose high alumina composition and smaller particle size allow its rapid dissolution and subsequent the precipitation of the hydration products [8]. However, the addition of sodium silicate in the mixtures inhibited the hydration delay of cement caused by the presence of ZnO. This aspect could confirm that the formation of the Zn(OH)_2_ phase is responsible for the hydration delay of cement at the initial setting time and that at pH >12 this phase does not form or precipitates in small amounts [47]; therefore, the initial setting time is delayed for minutes. 

In addition, at a pH > 12, the compounds Zn(OH)_3_^−^ and Zn(OH)_4_^2−^ are preferentially formed, which have no influence on the delay of cement hydration [48]. This suggests that ZnO reacts with sodium silicate, forming an insoluble Zn silicate gel, which is subsequently transformed into calcium zincate. Once all the Zn has been incorporated into the CaZn_2_(OH)_6_ 2H_2_O phase, the additional calcium product of the dissolution of the OPC particles allows the nucleation and growth of the C-(N)-ASH gel [45].

### 2.2. Compressive Strength

**Figure 1:**
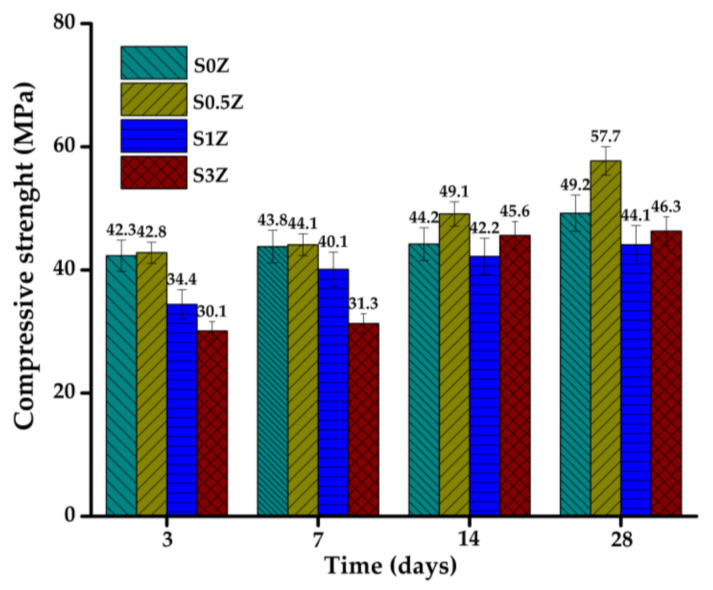
Compressive strength of samples after 3, 7, 14, and 28 curing days.

Figure 1 presents the compressive strength results of the samples studied as a function of curing time. The results show that the mixture with 0.5 wt% ZnO reached the highest compressive strength values at all curing ages. These superior values of compressive strength can be attributed to the filling effect promoted by the presence of ZnO [49], as well as the fact that it did not delay the hydration of the pastes nor the development of compressive strength at early ages. 

The compressive strength values of the ternary pastes with additions of 1 and 3 wt% ZnO were lower compared to those of the S0Z and S0.5Z samples, mainly after 3 and 7 curing days. Although in the setting time tests the effect of the addition of ZnO in the ternary pastes containing sodium silicate was not significant, a delay in the development of compressive strength at early ages was observed. This can be attributed to the fact that the phases responsible for the delay in the hydration of the mixtures in the first stages were not very significant. However, calcium zincate continues to form as long as there is sufficient Ca^+^ in the pore solution from dissolution of the cement particles and Zn(OH)_4_^2−^ species formed during the mixing of the ZnO in the alkaline solution, delaying the hydration of samples in the acceleration stage of hydration and, therefore, in the development of early strengths.

### 2.3. X-ray Diffraction

**Figure 2:**
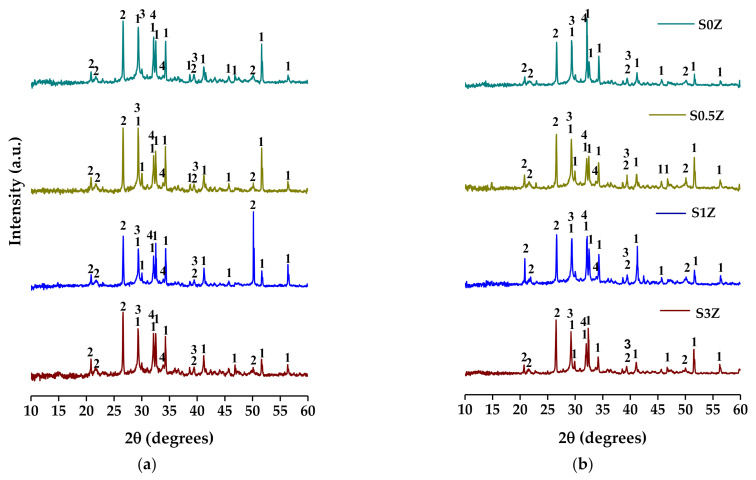
X-ray diffraction patterns of samples studied: (**a**) 7 and (**b**) 28 curing days. ^1^C_3_S y/o C_2_S, ^2^cuarzo, ^3^Na_2_SO_4_, and ^4^CaCO_3_.

Figure 2 shows the diffraction patterns of samples studied after 7 and 28 curing days. The results indicate that the phases present in these pastes are C_3_S, C_2_S, and quartz, which correspond to the main anhydrous phases of OPC and SCM, respectively, as well as the Na_2_SO_4_ and CaCO_3_ phases. In addition, the presence of a halo can be observed in a 2θ range between 20° and 35°, characteristic of C-A-S-H and N-A-S-H gels [50]. These results indicate that sodium silicate hinders the dissolution of OPC components [46] and inhibits the precipitation of C-S-H, CH, and ettringite due to the preferential formation of CaCO_3_ and Na_2_SO_4_ phases [24].

The above explains why the studied pastes added with alkali solution do not reach the highest values of compressive strengths after 7, 14, and 28 curing days. However, a strongly alkaline solution promotes the rapid formation of the hydration products of the pozzolans used (N-A-S-H, C-A-S-H, and (N,C)-A-S-H) and the development of higher early compressive strength that is obtained in traditional cement without high reactive SCM.

### 2.4. Fourier Transform Infrared Spectroscopy

**Figure 3:**
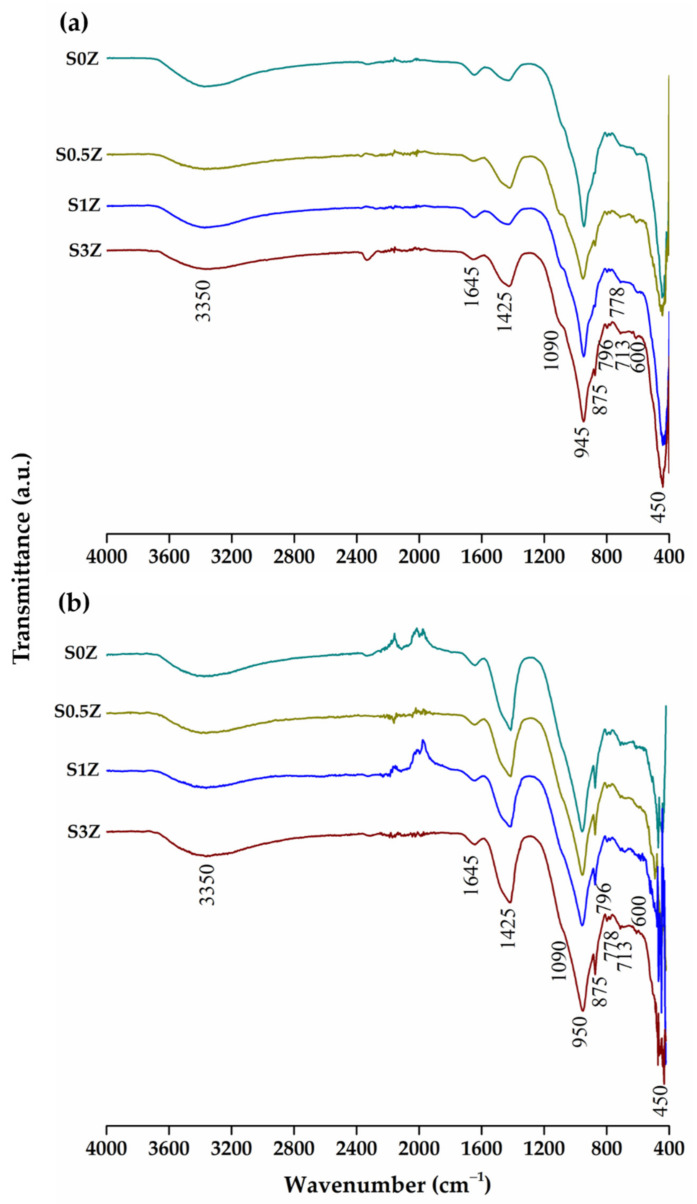
Fourier transform infrared spectra of pastes after (**a**) 7 and (**b**) 28 curing days.

Figure 3 presents the FTIR spectra of the studied pastes added with ZnO after 7 and 28 curing days. In all the spectra, the OH stretching and bending bands can be identified at 3350 cm^−1^ and 1645 cm^−1^, respectively, associated with chemically bound water and free water present in the pastes, as well as the CO tension band at 1425 cm^−1^, accompanied by bending bands at 875 cm^−1^ and 713 cm^−1^, associated with the presence of carbonates [51]. In addition, the Si-O stretching bands can be identified at 1090 cm^−1^, 796 cm^−1^, and 778 cm^−1^, associated with the compounds present in the pozzolans, and a Si-O stretching band at 950 cm^−1^, which could be associated with the precipitation of CSH, but mainly with the polymerization of the silicates present in the pozzolans used [42,52]. This could be corroborated due to the presence of the Si-O bending band at 450 cm^−1^ associated with the anhydrous calcium silicates of cement and the absence of the O-H tension band at 3642 cm^−1^, associated with calcium hydroxide (CH). The results obtained by this technique corroborate the information previously analyzed by XRD and the compressive strength tests of the analyzed pastes, where it was possible to identify that the pastes are partially hydrated and that the associated anhydrous phases of the OPC continued to be present.

### 2.5. Thermogravimetric Analysis and Differential Scanning Calorimetry

**Figure 4:**
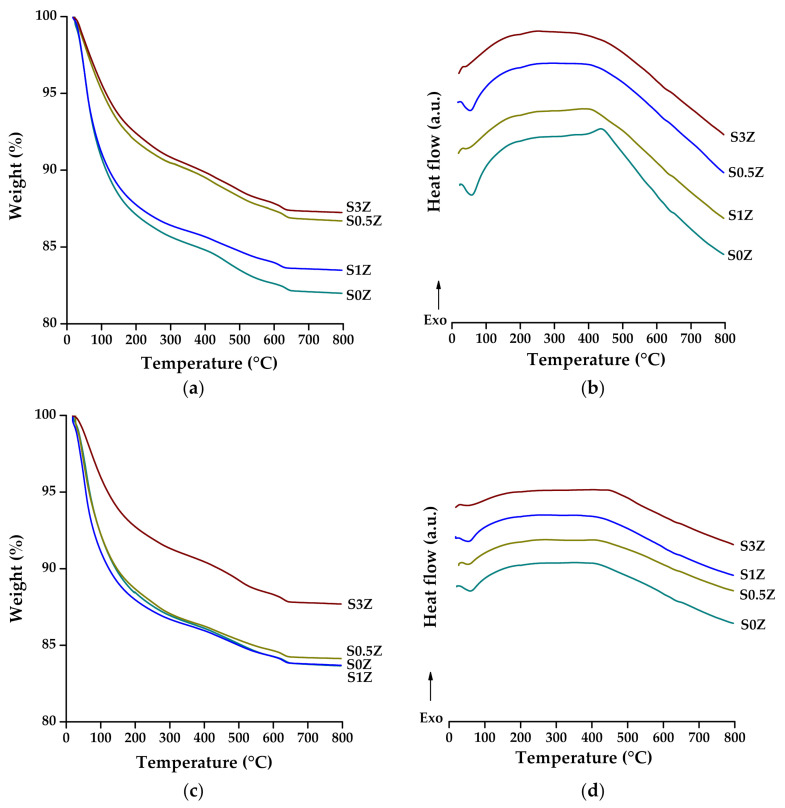
TGA-DSC of pastes added with ZnO: (**a**) TGA and (**b**) DSC at 7 days; (**c**) TGA and (**d**) DSC at 28 days.

Figure 4 and Table 2 show the weight loss of the pastes analyzed after 7 and 28 curing days, as well as the DSC curves associated with these losses. The first part of the weight loss may be affected by incomplete drying of the samples, but from 80 to 800 °C, the main loss identified is associated with molecular water of the hydrated products of the pastes (N-A-S-H, C-A-S-H, and (N,C)-A-S-H); the weight loss due to decarbonation of calcite was taken as the loss from 600 to 780 °C [53]. The DSC curve around 100 °C shows an endothermic peak attributed to the reactions associated with these losses. Neither a weight loss from 400 to 500 °C nor its endothermic peak associated with CH, usually found in PC pastes, was identified. The above confirms that the addition of sodium silicate inhibits the formation of OPC hydration products, as could also be observed in the results obtained by XRD and FTIR, where the phases and vibrations of the bonds associated with the anhydrous compounds of the OPC were observed, and the phases and vibrations of the OH bonds associated with CH were not identified.

### 2.6. Scanning Electron Microscopy 

**Figure 5:**
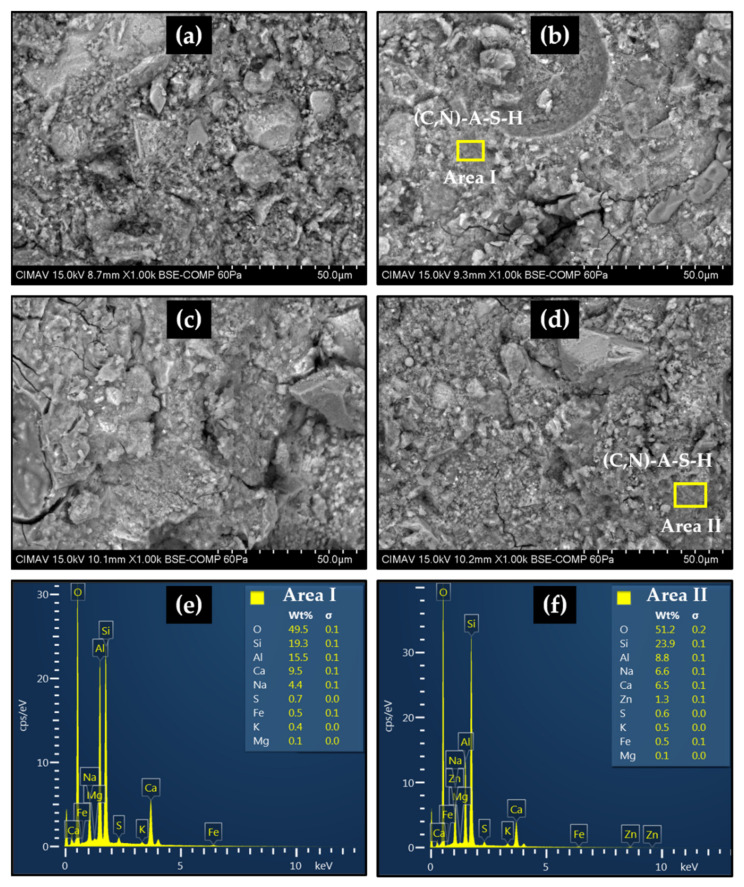
Scanning electron micrographs of pastes after 28 curing days: (**a**) SOZ; (**b**) S0.5Z; (**c**) S1Z; (**d**) S3Z; (**e**) EDX area I, (**f**) EDX area II.

Figure 5 shows BSE-SEM micrographs of samples added with ZnO analyzed after 28 curing days and EDX of the selected areas. The microstructure of all the pastes presents a heterogeneous and poorly compacted matrix, made up of anhydrous particles, such as calcium silicates and quartz. The presence of gels composed of O, Si, Al, Na, and Ca can also be seen, which could be mainly associated with the (C,N)A-S-H gel, as suggested by the results of the EDX analysis. In addition, some microcracks and different types of pores can be observed, including capillary pores larger than 50 nm, called macropores, which could have a negative influence on the development of mechanical strength. This confirms that the use of large quantities of SCM are necessary to obtain highly efficient behavior to hybrid cement due to the detrimental effect that sodium silicate has in PC.

## 3. Materials and Methods

### 3.1. Materials

**Figure 6:**
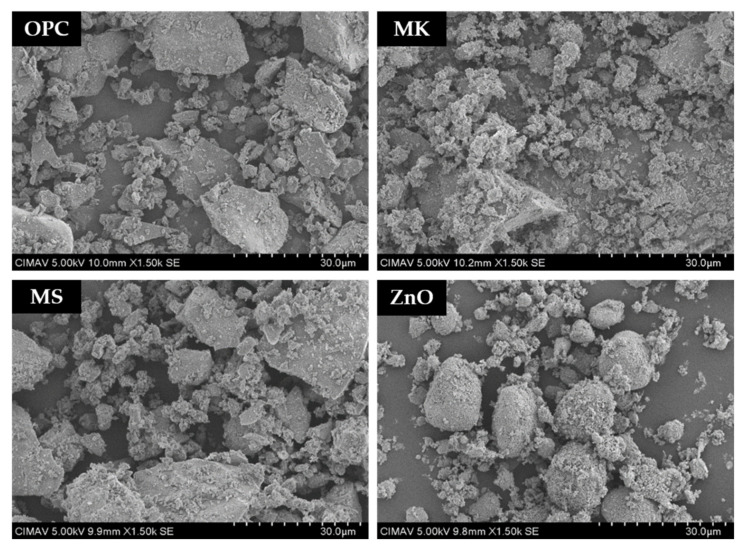
SE-SEM micrographs of precursor materials.

The raw materials to produce the pastes studied are Ordinary Portland Cement (OPC), two manufactured pozzolans by the calcination of kaolinite and ignimbrite, Metaforce^®^ (MK) and Microsilex^®^ (MS), respectively, supplied by GCC, and sodium metasilicate pentahydrate, Pentasil^®^, distilled water, and ZnO supplied by FagaLab, Mexico, with a purity of 99% and an average particle size of 11.5 μm. Scanning electron microscopy (SEM) was used to observe the morphology of raw materials; a JEOL JSM-7401F microscope (JEOL Ltd., Tokyo, Japan) operated at 5 kV and 150 μA was used. Micrographs of these materials showed angular particles approximately 1–30 µm in size (Figure 6).

Complementary analysis was made by X-Ray Fluorescence (XRF) in order to determine the oxide chemical composition of the materials; an Epsilon 3XLE energy dispersive X-ray fluorescence spectrometer developed by Panalytical (Malvern, UK) was used. For the analysis, samples were compacted into a tablet of approximately 5 g. The average particle size (APS) of the raw materials was determined using a CILAS 1180 laser particle size analyzer (CILAS, Orleans, France). Loss on ignition (LOI) was determined following the ASTM C 114 standard. The results are shown in Table 3. 

**Figure 7:**
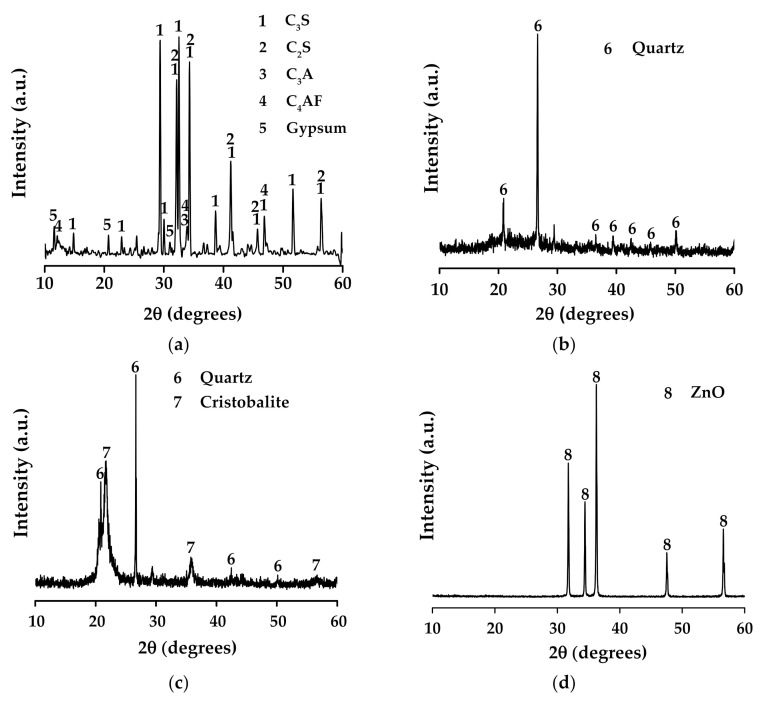
X-ray diffraction patterns of precursor materials: (**a**) OPC, (**b**) MK, (**c**) MS, and (**d**) ZnO.

For the XRD analysis, a Panalytical X’Pert PRO diffractometer with X’Celerator detector was used, under a Cu Kα radiation (λ = 0.15418 nm), at 40 kV and 30 mA in the 2θ range of 10–60°; the step and acquisition time were 0.02° and 5 s, respectively. The XRD results (Figure 7) confirm that the predominant phases on OPC are tricalcium silicate (C_3_S) (PDF 01-086-0402), dicalcium silicate (C_2_S) (PDF 00-001-1012), tricalcium aluminate (C_3_A) (PDF 00-008-0006), tetracalcium aluminoferrite (C_4_AF) (PDF 01-074-0803), and gypsum (PDF 00-021-0816). For the MK and MS, the main peaks correspond to quartz (PDF 01-078-1252), in addition to cristobalite (PDF 01-082-0512) for the latter. Finally, XRD traces of ZnO were indexed to PDF-01-079-2205.

### 3.2. Preparation of Pastes

The mixing proportions of hybrid pastes with ZnO additions of 0 wt% (S0Z), 0.5 wt% (S0.5Z), 1 wt% (S1Z), and 3 wt% (S3Z) of solids were 60 wt% OPC, 30 wt% MK, and 10 wt% MS, with a liquid/solid ratio of 0.35. Before mixing, sodium metasilicate pentahydrate was added to the water and stirred mechanically for 4 h, and then the ZnO was added and stirred for another 2 min. Subsequently, the pastes were prepared according to the ASTM C 305 standard and samples were cast in iron molds and wrapped in plastic film to rest for 24 h. After this time, the samples were unmolded and cured at 30 ± 2 °C in a high relative humidity environment until the day of the compressive strength tests.

### 3.3. Tests Conducted

The setting times of pastes were determined according to the ASTM C 191 standard using a manual Vicat apparatus. Compressive strength tests were conducted using an INSTRON 600DX universal testing machine (INSTRON, Norwood, MA, USA) after 3, 7, 14, and 28 curing days. 

For the XRD, FTIR, and TGA-DSC characterization, the preparation of samples was carried out after performing the mechanical tests after 7 and 28 curing days. Fragments of samples were milled until a fine powder was obtained. In order to stop the hydration process of pastes, the powders were washed with acetone and ethanol and dried in an oven at 60 °C. 

The identification of hydrated phases present in the pastes was evaluated by XRD in a D8 Advance diffractometer (Bruker, Billerica, MA, USA) coupled with a copper (Cu) lamp (λ = 0.15406 nm), in the 2θ range from 10° to 60° with a scanning step of 0.02°/min. A complementary study of FTIR was recorded over a range of 4000–400 cm^−1^ with an FTIR spectrometer Bruker Alpha I. The TGA-DSC studies were conducted to obtain the weight loss of the hydrated products using a TA model Q600 instrument. The analysis was performed using a rate of 10 °C/min up to 800 °C.

For the analysis of the microstructural properties of pastes, fragments of samples tested after 28 curing days were submerged in acetone for 24 h and dried in an oven at 60 °C for 24 h. Finally, the samples were placed on a graphite tape and an ultra-fine gold coating was applied on the sample surface to ensure the electron conduction. For this study, a JEOL microscope model JSM-7401F was used, which was operated at different acceleration voltages and working distances; both backscattered (BSE) and secondary electron (SE) detectors were used. Energy-dispersive X-ray (EDX) analyses were conducted in regions of interest. 

## 4. Conclusions

According to the results presented, it is proposed that the addition of ZnO in hybrid cements delays the setting time of pastes up to 39 min and high mechanical properties at early age can be obtained without additional temperature, as is the case with alkaline-activated materials. However, the use of large quantities of SCM are necessary to obtain a highly efficient behavior to hybrid cement due to the detrimental effect that sodium silicate has in PC.

Important conclusions from this investigation are the following:

The use of an alkaline solution in the mixture reduces the delay effect in the hydration of cement, which is caused by the addition of ZnO. This proves that the Zn(OH)_2_ phase is the main responsible for the delayed effect on hydration of PC pastes and that it is barely formed when the pH of the mixing water is higher than 12.

The maximum delay of the initial and final setting time of samples was 22 and 39 min, respectively.

The addition of 0.5 wt% ZnO has a positive effect on the mechanical properties of the pastes by increasing the compressive strength values at all curing ages.

Although the presence of ZnO in hybrid pastes with the addition of sodium silicate did not have a significant effect on the delay of setting times, the addition of 1 and 3 wt% ZnO decreased the compressive strength values at early ages by 30%.

The alkaline solution promotes a rapid hydration of SCM, developing high values of early compressive strength. However, it has a detrimental effect on the OPC hydration products and consequently on the development of later mechanical strengths; this causes an insignificant increase (15%) with a curing time from 3 to 28 days. 

The microstructure of hybrid pastes presents partially hydrated matrices associated with hydrated products as (C,N)-A-S-H and unhydrated particles, mainly associated with PC due to the detrimental effect of alkaline solution on PC. However, no evident microstructural evolution was experienced regardless of the ZnO added to the pastes.

The addition of alkali solution in ternary pastes with ZnO can be of interest in cementitious materials that require the use of ZnO additions higher than 0.5 wt% and a rapid setting.

## Figures and Tables

**Table 1:** Setting time (min) of samples.

Sample	Initial Setting Time	Final Setting Time
S0Z	8	16
S0.5Z	11	25
S1Z	23	32
S3Z	30	55

**Table 2:** Weight loss (%) of samples.

Sample	7 Curing Days	28 Curing Days
S0Z	5.4	5.6
S0.5Z	5.2	5.4
S1Z	4.9	4.6
S3Z	5.1	5.0

**Table 3:** Chemical oxide composition (wt%), LOI (g), and APS (µm) of precursor materials.

Sample	SiO_2_	Al_2_O_3_	Fe_2_O_3_	CaO	MgO	Na_2_O	K_2_O	SO_3_	LOI	APS
OPC	20.7	4.3	2.9	63.7	2.3	0.2	0.1	2.9	2.7	13.6
MK	55.5	30.8	1.9	6.7	0.3	0.8	0.4	0.7	2.9	10.9
MS	82.2	6.9	1.3	5.7	0.1	0.3	0.1	0.9	3.0	12.2

## Data Availability

Not applicable.

## Sample Availability

Samples of the hybrid pastes produced in this investigation are available from the authors.
